# Disturbance-specific social responses in long-finned pilot whales, *Globicephala melas*

**DOI:** 10.1038/srep28641

**Published:** 2016-06-29

**Authors:** Fleur Visser, Charlotte Curé, Petter H. Kvadsheim, Frans-Peter A. Lam, Peter L. Tyack, Patrick J. O. Miller

**Affiliations:** 1 Kelp Marine Research, Loniusstraat 9, 1624 CJ, Hoorn, The Netherlands; 2 Behavioural Biology Group, Leiden University, PO Box 9505, Leiden, 2300 RA, The Netherlands; 3 CEREMA–DTer Est, Acoustics Group, F-67035, Strasbourg Cedex 2, France; 4 Norwegian Defence Research Establishment FFI, Maritime Systems Division, NO-3191, Horten, Norway; 5 Acoustics and Sonar, TNO, PO Box 96864, The Hague, 2509 JG, The Netherlands; 6 Sea Mammal Research Unit, University of St. Andrews, St. Andrews, Fife, KY16 8LB, UK

## Abstract

Social interactions among animals can influence their response to disturbance. We investigated responses of long-finned pilot whales to killer whale sound playbacks and two anthropogenic sources of disturbance: tagging effort and naval sonar exposure. The acoustic scene and diving behaviour of tagged individuals were recorded along with the social behaviour of their groups. All three disturbance types resulted in larger group sizes, increasing social cohesion during disturbance. However, the nature and magnitude of other responses differed between disturbance types. Tagging effort resulted in a clear increase in synchrony and a tendency to reduce surface logging and to become silent (21% of cases), whereas pilot whales increased surface resting during sonar exposure. Killer whale sounds elicited increased calling rates and the aggregation of multiple groups, which approached the sound source together. This behaviour appears to represent a mobbing response, a likely adaptive social defence against predators or competitors. All observed response-tactics would reduce risk of loss of group coordination, suggesting that, in social pilot whales, this could drive behavioural responses to disturbance. However, the behavioural means used to achieve social coordination depends upon other considerations, which are disturbance-specific.

Disturbance responses take time and energy away from other critical activities, such as foraging and reproduction. Moreover, reactions such as flight further increase energetic cost^1^. These costs must be balanced against the associated risk, which, in the case of predation, can be broken down into risk of detection and risk of capture^2^,^3^,^4^. Most bird species, for example, don’t initiate a flight response until a threat has approached to a certain critical range^5^. Many species have evolved abilities to classify different predators and different risks of predation, giving rise to the existence of predator- and risk-specific response tactics^2^,^6^,^7^. Furthermore, the nature and magnitude of responses may vary considerably across environmental (e.g. proximity to protective cover^1^), social (e.g. group size^8^) and individual contexts (e.g. body-condition^9^). Thus, the extent to which an animal changes its baseline behaviour should scale with the risk associated to the disturbance^10^, and natural factors which influence responsiveness^11^.

In social animals, individual cost-benefit assessment and choice of response tactics to external stressors are influenced by those of group members^12^. For example, alarm calls may increase the ability of groups to detect predators early, indicate risk of predation and coordinate responses within a group^13^. One of the major benefits of group living is the capability for group members to respond in a coordinated fashion, *i.e*. individuals can coordinate actions to detect and fend off threats, with significant reduction of disturbance-associated risk and cost to individuals^14^. Hence, for social species, sociality may form a major determinant of their choice of response tactic.

Toothed whales are social mammals that rely mainly on sound to acquire prey, navigate and coordinate with conspecifics, which makes them particularly vulnerable to noise disturbance^15^,^16^. Sound sources such as naval sonar and vessels can lead to strong behavioural responses, including cessation of foraging and area avoidance, which have the potential to accumulate to population-level effects^17^,^18^,^19^,^20^. Nonetheless, the biological significance of toothed whale responses to different disturbances remains poorly understood. This is partly due to large differences in the nature and magnitude of the response within and between species^21^,^22^,^23^. Furthermore, most toothed whale response studies have reported on movement or diving behaviour of individuals (e.g.^24^,^25^). Here, we focus on social aspects of behavioural responses to disturbance.

Long-finned pilot whales (*Globicephala melas*) are social toothed whales that live in stable matrilineal groups within larger aggregations^26^. Groups cooperate in defence against predators or competitors and coordinate the timing of their foraging behaviour^27^,^28^,^29^. Most cetaceans are highly vocal^30^ and exhibit auditory-mediated responses to heterospecific as well as man-made sounds^27^,^31^,^32^. Compared to most other cetaceans tested, pilot whales show relatively high threshold levels for behavioural responses from acoustic disturbance by naval sonar^20^,^22^,^23^,^24^,^25^,^33^.

To explore the response tactics of social toothed whales and the biological significance of their behavioural responses, we investigated the type and magnitude of long-finned pilot whale responses to two sources of anthropogenic disturbance (tagging effort, naval sonar) and compared these to their responses to a natural heterospecific disturbance, playback of the sounds of killer whales. Attempts to tag a whale involve following a group and repeatedly approaching one or several individuals to tag them. This situation is similar to cases when a potential threat has already detected a group and is closely approaching it, where the group response serves to reduce negative consequences of the hazard. By contrast, exposure to a distant sonar may stimulate responses designed to avoid detection and closer approach. We used playbacks of killer whale (*Orcinus orca*) sounds to simulate the presence of a potential predator or competitor. This biological stimulus reveals which action animals will undertake when perceiving a natural threat to which behavioural responses have been shaped by evolution^10^. This aids the interpretation of the level and biological relevance of responses to anthropogenic stimuli that may represent relatively novel disturbances.

## Results

**Figure 1:**
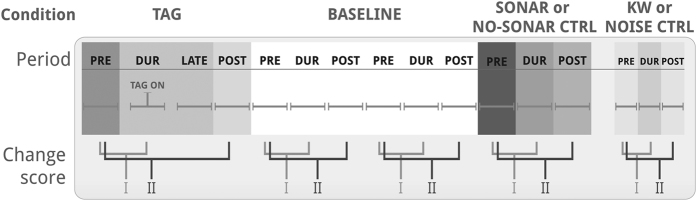
Experiment-timeline design. Experiments consisted of one to four phases (tagging (TAG), baseline, sonar exposure and killer whale playback (KW)), including 6 experiment conditions. The number of sessions within the baseline, sonar and killer whale playback phases varied between experiments (Table 1). Two sets of change scores were calculated: I) change in behaviour between during and pre-exposure periods (PRE_DUR) and II) change in behaviour between post- and pre-exposure periods (PRE_POST). LATE = final 30 minutes of tagging phase. CTRL = control

**Figure 2:**
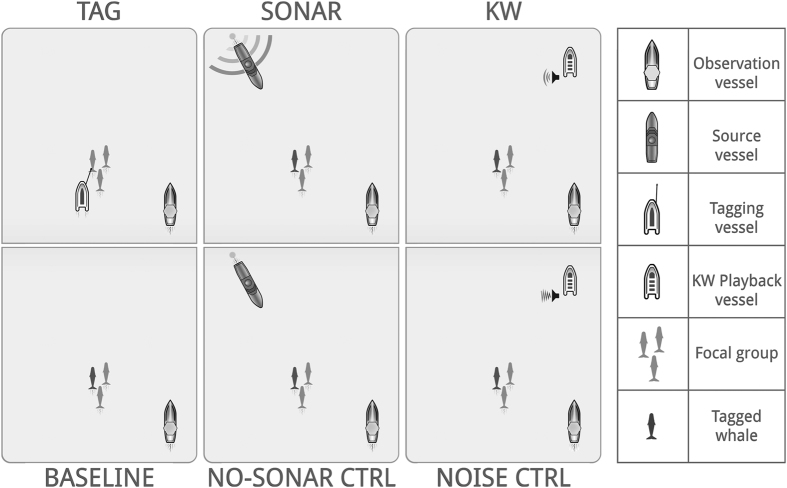
Schematic representation of experimental approach (not drawn to scale). Observation vessel (29 m): aimed to maintain distance of 100–400 m to the focal group, representing a consistent factor in all experiment conditions; Source vessel (55 m): direct approach and signal transmission from 6–8 km at 3–4 ms^−1^. At 1 km distance, vessel course is locked. Approach and transmission continued until ~5 minutes after passing the focal group (closest approach: 40–560 m; mean = 250 m^13^;). Sonar: transmission of sonar signals. No-sonar control (ctrl): no transmission; Tagging vessel: consistent close approach aiming to apply a tag; Killer whale playback vessel: stationary, in front and to the side of focal group travel path (2400 ± 943 m^27^;). KW: transmission of killer whale vocalisations. Noise ctrl: transmission of broadband noise.

We conducted experiments with 14 tagged and 2 non-tagged pilot whales and their associated groups (Fig. 1, Table 1). Experiments included 6 conditions (Fig. 2): we tested 15 focal whales and their groups with tagging effort, 10 with baseline, 5 with sonar exposure and/or no-sonar control and 5 with killer whale playbacks (KW) and/or noise control (Table 1). Experiment durations ranged from 2 to 21 hours (h) (mean ± SD = 8.5 ± 5.7 h).

**Figure 3:**
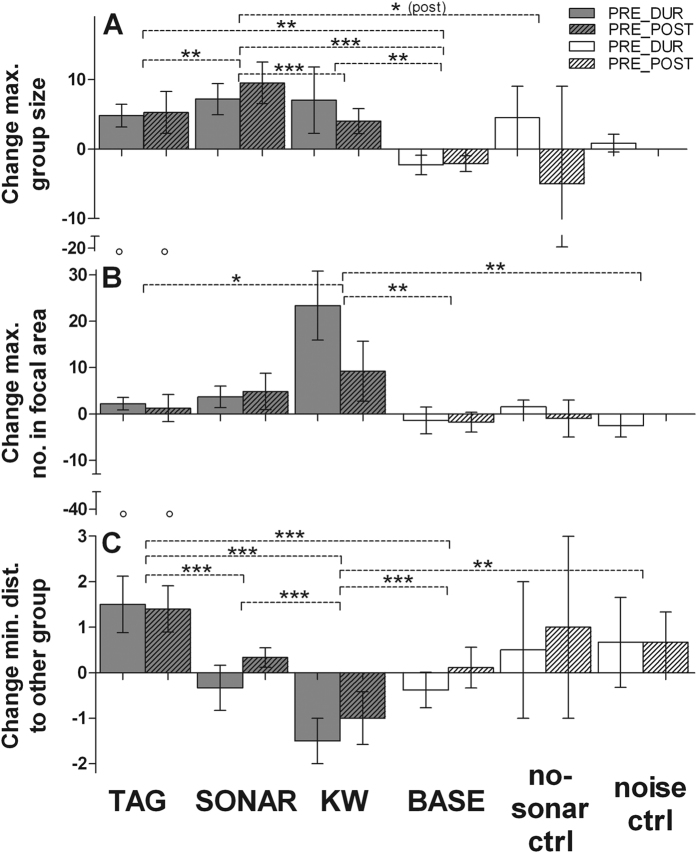
Changes in the aggregation behaviour of long-finned pilot whales in response to the 3 disturbance conditions (tagging effort (TAG), sonar exposure and killer whale playback (KW); grey bars) in comparison to baseline (BASE), the no-sonar control (ctrl) and the noise ctrl (white bars). Positive or negative change scores respectively indicate an increase or decrease in (**A**) maximum (max.) group size, (**B**) maximum number (no.) of individuals within 200 m and (**C**) minimum (min.) distance to the nearest other group. PRE_DUR: difference in behaviour between during and pre-exposure period. PRE_POST: difference in behaviour between post- and pre-exposure period. Error bars indicate SE, open circles indicate outliers Dashed lines indicate significantly different changes between conditions at *p* *<0.05, **<0.01 or ***<0.0001. (post) indicates that a significant difference from the pre-exposure period was found only for the post exposure period.

**Figure 4:**
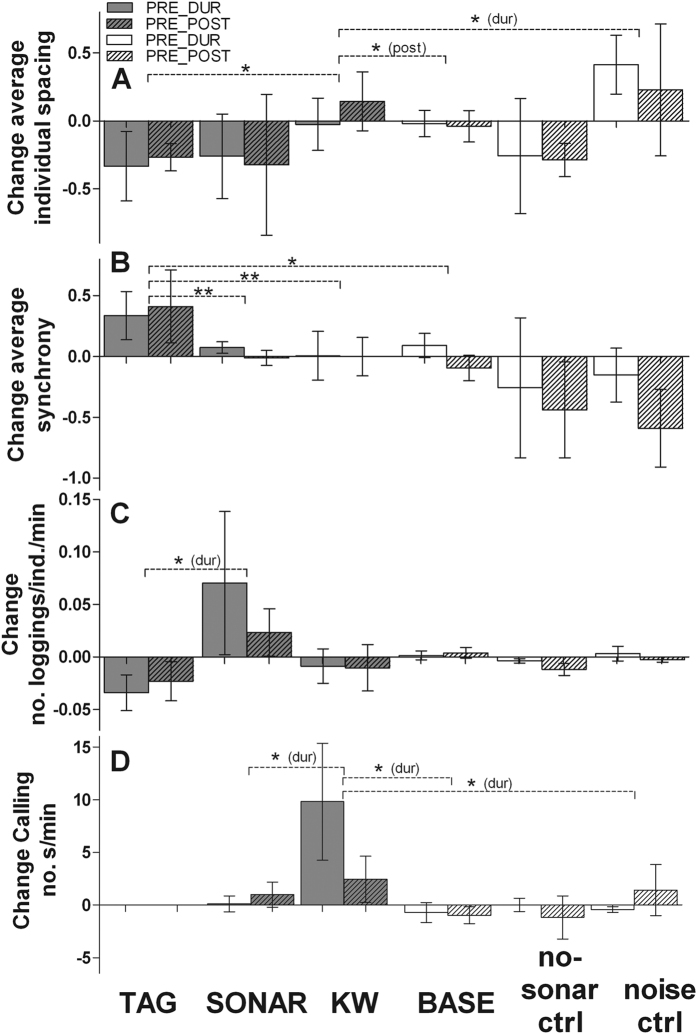
Changes in the cohesion, logging and calling behaviour of long-finned pilot whales in response to the three disturbance conditions. Positive or negative change scores respectively indicate an increase or decrease in (**A**) average spacing between individuals, (**B**) average synchrony of surfacing, (**C**) number (no.) of loggings individual^−1 ^minute^−1^ and (**D**) the number of seconds per minute that whales produced calls. Figure description as in Fig. 3. As we could not collect pre-tagging data for vocalisations (no tag deployed), changes in calling behaviour could not be assessed for tagging effort.

Group-level and vocal behaviours were generally stable over the duration of our experiment periods, as indicated by the low degree of change during baseline conditions (pre, during (DUR) and post periods were simulated for the baseline phase; Figs 1, 3, 4). Experiment condition had a significant effect on the change scores for all parameters (Generalised Estimating Equations (GEEs), all: p ≤ 0.002; Table S1). Comparison of the change scores between the 6 conditions revealed strong contrasts in responses to tagging, sonar exposure and killer whale playbacks (KW), which were confirmed by the GEE analyses.

### Aggregation

We observed a significant increase in pilot whale group size during all three disturbance types, tagging, sonar exposure and KW, relative to baseline periods (GEEs, all: p ≤ 0.007; Fig. 3A, Table S2). Average group size (±SD) during baseline was 10.1 ± 5.2. The strongest increase in group size was observed during and post sonar exposure, with an average increase in maximum group size of 7 individuals during exposure, which increased even more during the post-exposure period. During tagging, the increase in group size was accompanied by a significant increase in the distance to other groups (1 to 2 range steps, 50–500 m per step; GEE, p < 0.0001; Fig. 3C, Table S2), relative to baseline conditions, with only rare occurrence of other groups within 100 m of the group targeted for tagging (6% of records vs. 29% during baseline). In 21% of tagging records, no other groups were present within the sighting range (1000 m), which was comparable to baseline (17%), but contrasted with sonar exposure (7%) and KW (8%). Pilot whale groups drew significantly closer together during KW (GEE, p < 0.001; Fig. 3B,C), leading to an average increase of 23 individuals in the focal area (average number in area ± SD during baseline = 15.3 ± 7.9). Transitions between deep and shallow diving resulted in a reduction in group size and the number of individuals in the focal area, with respect to periods that only encompassed periods of shallow diving. However, effects of diving state were smaller than those recorded for experiment condition (Table S2).

### Coordination, Surface Activity & Calling

During tagging effort, pilot whale focal groups increased their surfacing synchrony, relative to baseline conditions (GEE, p = 0.01; Fig. 4B; Table S2), with more occurrences of high synchrony (17% of records) and of very tight individual spacing (48%) than during baseline (12% and 15%). Also, there was a tendency for fewer loggings in the focal group (Fig. 4C). This contrasted with a tendency for an increase in the number of loggings during sonar exposure (Fig. 4C, Table S2). Individual spacing was higher after, but not during, KW, relative to baseline and noise control (GEE, p ≤ 0.03; Table S2), whereas there was a tendency for reduced individual spacing during tagging effort and sonar exposure. For individual spacing, response to the noise control was more pronounced than recorded for KW (Fig. 4A). In addition to experiment condition effects, changes in individual spacing also resulted from changes in diving state (Table S2).

Calling was recorded during an average (±SD) of 6 ± 7% of time during baseline (range: 0–44.7%). Calling was higher during KW (recovering in post-exposure), relative to baseline and noise control (GEE, p < 0.05; Fig. 4D, Table S2). This increased calling coincided with increased numbers of animals near the tagged whale during and post KW (Fig. 3A,B).

### Silence & Vocal activity

Whales were silent (interval between consecutive vocalisations >24.5 s) on average (±SD) 40 ± 33% of the time during baseline. In total, 12 silent experiment periods (90–100% of time in period silent) were recorded. Silent periods were rarely recorded in baseline (5 of 43 DUR and POST periods; 12%) and sonar (1 of 24; 4%), and were absent in KW (0 of 8). However, silence occurred relatively regularly DUR and POST tagging (5 of 24; 21%), representing nearly 50% of all silent periods recorded.

Highly vocal experiment periods (90–100% of time vocal; N = 27) were more common than silent experiment periods. Highly vocal periods occurred relatively regularly in baseline (13 of 43 simulated DUR and POST periods; 30%). In total, 10 out of the 14 highly vocal periods were recorded POST exposure; records in DUR were rare (12% of DUR vs. 29% of POST periods). Highly vocal periods were most common post sonar (4 of 12 periods; 33%) and post noise control (3 of 3 periods). Whales were highly vocal during 1 of 12 sonar exposure periods, but in this case, the group was already highly vocal during pre-exposure.

## Discussion

Our results show that the type and magnitude of long-finned pilot whale (*Globicephala melas*) social and vocal responses differed across three disturbance conditions. The active grouping together and approach response of pilot whales to a natural disturbance (killer whale (*Orcinus orca*); similar to a ‘mobbing’ response^27^) did not occur when they were exposed to two anthropogenic disturbances (tagging effort and naval sonar exposure). Instead, tagging effort elicited a response where group members became more synchronous, more distant from other groups and reduced vocal activity later in the exposure, while sonar exposure induced a response where animals came together at the surface.

Pilot whales groups use social defence strategies after detecting a natural threat, such as the presence of potential predators or competitors^27^,^28^. Hearing killer whale vocalisations elicited a clear response in the pilot whales, inducing a strong aggregation of individuals and groups, while increasing or maintaining social calling rates. Tagged whales and their groups, as well as most other groups in the aggregation, were attracted to the playback speaker and aggregated near the sound source^27^. Hence, pilot whales seemed to respond to killer whale presence as a disturbance that could be safely investigated and effectively fended off by mobbing.

Tagging effort involved consistent noisy and repeated targeted pursuit by an agile vessel aiming to make physical contact (apply a tag). Pilot whale responses during tagging contrasted with those observed during killer whale playbacks (broadcasted from a vessel drifting with its engine turned off). During tagging, whales increased their group size and within-group synchrony, and showed a tendency to reduce the spacing between group members and the degree of surface resting (logging). Whereas the targeted group could merge with its nearest neighbour, other nearby groups in the aggregation did not approach and many even moved further away. We could not assess changes in vocal behaviour relative to pre-exposure for the tagging condition, because the tags had to be attached to record calls. However, call rates were significantly reduced comparing the period directly following tag deployment and the last 30 minutes of tagging effort (LATE; Fig. 1), to baseline conditions (GEE; N = 9 experiments, change score DUR_LATE tagging: estimate ± SD = −3.3 ± 1.3, p = 0.013). Higher rates of social calling (12%) were recorded in the period directly following tag placement with respect to the final 30 minutes of tagging and post-tagging (8 and 7%), during which they could become silent.

It is unlikely that the whales we tagged had been exposed to tagging effort prior to our study. However, some vessel and whale watching activity exists in our research area, and whales may have been exposed to repeated approaches by small boats. Whereas aggregation and mobbing can be successful when dealing with killer whales^28^, it does not seem an effective strategy when aiming to avoid pursuit by a small mobile boat that repeatedly approaches the group. The observed tactic more closely represents an evasion response to a potential threat that has detected the group, has engaged in pursuit, and that cannot reliably be fended off.

Marine mammals could perceive powerful sounds such as naval sonar signals as a risk of discomfort due to the loudness of the sound exposure, as a risk for reduced coordination (masking, disorientation), or as a potential threat^10^,^15^. Most cetacean species exposed to sonar show strong horizontal or vertical avoidance responses, effectively distancing them from the sound source^20^,^24^,^34^. In pilot whales, avoidance responses to sonar happened at relatively high levels, compared to those in experiments with killer whales, beaked whales (Ziphidae) and minke whales (*Balaenoptera acutorostrata*)^20^,^23^,^25^,^33^,^34^. Rather than showing a vertical avoidance response, during sonar exposure pilot whales showed a preference for shallow diving depths (<35 m^35^). The transition from deep to shallow diving did not reduce the overall received levels of sonar^36^, which means that this response tactic was not primarily aimed at reducing received level. However, when the sonar was very close, some whales surfaced in synchrony with the loud sonar signals, potentially reducing sound exposure levels^36^. As whales did not prefer to be shallow diving during the no-sonar control runs of the source vessel, this response is specific to the sonar signals and not caused by the approaching vessel.

Sonar signals elicited a response that led pilot whales to increase group size and spend more time at the sea surface with their group members. Sonar exposure did not elicit changes in social calling rates, or a silencing response, but sometimes elicited mimicry of the sonar signals^32^. Together with absence of support for increased within-group cohesion or synchrony, in the sonar exposure setting of our experiments (one approaching source with escalating received levels), representing a potentially threatening scenario, these results suggest that sonar signals are not perceived as a sound source from which pilot whales *a priori* want to get away, or hide from. Instead, it may be a response where group members form larger groups to prepare for horizontal avoidance without losing social cohesion, or to prepare for social defences against threats, such as social mobbing. The aggregation response may also suggest that whales perceive the distant approaching loud sound as a potential risk of decreased coordination (vocal masking, disorientation). For example, following deep dives during which they can be several 100s of m apart, pilot whales use vocalisations to relocate group members^37^. Increasing levels of sonar may interfere with this process, leading whales to choose to aggregate more at the surface where they can also use visual cues to locate group members. The suggestion that there is a risk of masking or reduced opportunity for vocal exchanges during sonar exposure may be supported by increased vocalising post exposure.

The contrasting responses to a natural threat and two types of anthropogenic stimuli indicate the ability for behavioural plasticity in response to different types of disturbance. Our results show that pilot whale responses to anthropogenic stimuli do not match the template of response to a known natural disturbance. Animals tailor their behavioural responses to different kinds of disturbance in their environment, specific to their perception of the level and kind of associated risk^10^. Accurate assessment of risk and appropriate behavioural response pose obvious fitness benefits and may be crucial for survival, for example giving rise to the existence of predator-specific response tactics^6^,^7^,^13^. The observed behavioural plasticity opens the question to what extent cetacean social responses to novel anthropogenic stimuli may be adaptive behaviours that effectively reduce the impact of disturbance.

Mobbing responses to killer whale sounds will have been shaped by natural selection, and appear to form a successful social defence against these predators/competitors at least under some circumstances^28^. Clearly, a mobbing-type response to tagging or sonar vessels would not only be ineffective, but also potentially risky for responding whales (risk of collision, larger number of whales exposed at higher intensity). Much study of anti-predator strategies focuses on avoidance strategies to prevent detection by a predator, and evasion strategies to get away from the predator once detected^38^. However, whales exposed to an approaching sonar source increased group size and spent more time at the surface, both of which should increase detectability. From the perspective of the human tag-boat driver, the best way for a group of whales to lose a trailing boat is to spread out, surface asynchronously, and/or increase dive duration. By contrast, the whales being followed increased group size and surfaced synchronously, with other groups moving farther away, making it easier for the boat to follow the target group. As with the killer whale playbacks, the behavioural responses to sonar and tagging do not have the features expected if they function to escape detection or repeated approach.

The unifying characteristic of the pilot whale social response to disturbance is to allow for individuals to remain closely associated with their group members (increase in group size) at the surface; individuals do not make the decision to disperse. This tactic, shared with some terrestrial social mammals^7^, allows pilot whales to maintain their social cohesion, and potentially to prepare to engage in either social defence tactics or group avoidance; *i.e*. stimulus-specific considerations ultimately determine the behavioural means used to maintain coordination. As it may take time to come together when dispersed, group-living animals with social defence strategies may need to anticipate using an early onset response, to ensure that they have reached a cohesive state at the time a group response needs to take place. This would be relevant for pilot whales, performing about 8 minutes of long deep foraging dives to several hundred of meters and generally occurring in aggregations spread out over a large area^29^, particularly for responses to an escalating sound source that can be heard tens of kilometres away. Given that pilot whales are unlikely to have time for a preparative response during close approach by a tagboat, the response to tagging effort may represent a later stage of response to anthropogenic disturbance, when whales have decided to increase their within-group cohesion and synchrony, and may become silent, while other groups may benefit from increasing distance from the targeted group, rather than joining it. This pattern may be comparable to the different response tactics employed during the successive stages of a hunt (search, detection, pursuit, attack)^4^, whereby the sonar and killer whale sound stimuli represent early stages (presence of ‘predator’, uncertainty of whether pilot whales have been detected or not), whereas tagging effort represents later stages, during which the pilot whales have been clearly targeted, and are being pursued.

Pilot whales are air-breathing marine animals that spend the majority of time shallow diving but tend to feed at depth^29^,^35^. If they have to recover physiologically from deep dives, coming together at the surface may be a response preceding an actual avoidance or other response that allows maximum flexibility to respond to a disturbance for which the level of risk and duration may be unknown. If the whales spend more time shallow diving and less time foraging, this response may come at an energetic cost, particularly during exposures that may extend over multiple hours or even days^31^.

This study showed that long-finned pilot whale groups exhibit stimulus-specific social and vocal responses to disturbance. Response tactics to killer whale sound playback matched those seen to actual killer whales. This mobbing response appears successful against killer whales but would not succeed against the vessel-based anthropogenic disturbances. The responses to a relatively distant approaching sonar are consistent with preparation for social defence against an unknown threat, while the responses to attempts to tag appear to function to protect group cohesion during evasion of repeated approaches. These observations suggest that pilot whales adapt their social responses to match the specific risks posed by the different forms of disturbance.

## Methods

Fieldwork was conducted off Northern Norway between 66°–70° N latitude in May/June of 2008, 2009 and 2010^39^. Three types of behavioural data were collected during experiments with long-finned pilot whales (*Globicephala melas*): social context at the surface from focal follow observations, and dive parameters and acoustic scene (including all whale vocalisations and acoustic stimuli) of tagged individuals. We consistently tracked one focal (tagged) whale and its associated group, allowing us to record the behaviour and social context of the focal individual throughout each experiment.

### Experimental protocol

Experiments comprised one to four consecutive phases: tagging, baseline, sonar exposure and playbacks of killer whale vocalisations (Fig. 1, Table 1). The sonar and killer whale playback phases also included controls, resulting in a total of 6 experiment conditions (Fig. 2). Experiments started with 30 minutes of pre-tagging behavioural observations of the focal group (Fig. 1). Then tagging effort was started to attach an archival suction-cup tag (DTAG^40^) to a whale in the focal group using a 6 m carbon fibre pole from a small boat. The boat approached the animals at slow speeds, while moving roughly parallel to the direction of their horizontal movement. Tagging effort was sustained until maximally 1 hour (h) after a first tag deployment, with the aim to deploy a second tag on a different individual in the same group, or could end without any tag being deployed (tagging effort duration: range = 1.4–3.3 h; mean ± SD = 2.2 ± 0.7 h), followed by 30 minutes of post-tagging data collection. During the ensuing baseline phase, the behaviour of the whales was recorded in absence of any of the stimuli (Fig. 2; duration: range = 0.8–10.6 h; mean ± SD = 4.4 ± 3.6 h). The baseline phase occurred post-tagging to enable use of tag data in our analysis. The 30-minute recovery period was chosen based on field visual observations of whales returning to pre-tagging behaviour in this period.

The sonar exposure phase consisted of two to four ~30 minute exposure sessions of sonar signals, or a no-sonar control. During sonar exposure, hyperbolic sweeps of 1 s duration were transmitted every 20 s (maximum source level (SL): 214 dB re 1μPa∙m) from a naval sonar source towed behind the approaching source vessel (Fig. 2). Transmissions started with a 10-minute ramp-up period, followed by full power transmissions. Hence, the sonar exposure represented the scenario of an escalating source (at the start of the transmission period) with an approach toward the location of the whales. The combination of initial approach with ramp-up could have appeared as a threatening rapid approach, but this phase of approach started 8 km from the whale, and is likely not equivalent to the rapid looming of a visual stimulus. The same pattern of vessel approach without transmissions was performed for the no-sonar control, to control for responses of the whales to the approaching source vessel. Sessions were conducted in an alternating order and separated by at least 55 minutes^39^.

The killer whale playback phase consisted of two to four 15-minute transmission sessions of recordings of killer whale vocalisations (KW) and/or a broadband noise control, separated by approx. 10 minutes, played in random order using an underwater speaker deployed from a small drifting boat^27^ (Fig. 2). The noise control, created from sections of the same killer whale recordings without vocalisations, was conducted to control for responses of the whales to any unspecific acoustic stimulus (SL of the KW and noise control stimuli was 140–155 dB re μPa∙m). The killer whale playback phase was separated from the preceding sonar exposure phase by at least 85 minutes (N = 2) or without preceding sonar exposure (N = 3; Table 1). See supplementary material for further details on sound stimuli.

### Tag data collection

We collected data from 14 tagged whales. Pressure sampled on the DTAGs was converted to diving depth using calibrated values, and decimated to a 5 Hz sampling rate^40^. In one case, depth data were collected from a second type of suction-cup attached tag, which recorded depth at 1-second intervals, but no sound^41^. Each diving record was divided into bouts of deep diving (foraging) and bouts of shallow diving (non-foraging), following the definition in Visser *et al*.^29^. When tag data were not available (pre-tagging, 2 focal follows without tag deployment), diving state was classified from the group-level parameters using the classification model in Visser *et al*.^29^.

Stereo sound recordings from the DTAGs (16-bit resolution at 96 or 192 kHz) were used to analyse changes in calling in the acoustic scene of the tagged whale, and to identify the presence of periods of silence and high vocality of the tagged whale and its nearby conspecifics. The start and end times of all vocalisations from the tagged whale and vocalising conspecifics that were audible and visible on a spectrogram (Blackman-Harris window, FFT window size: 4096 points, overlap: 75%; Adobe Audition 2.0) were marked by two independent observers. All sounds were classified by perceived signal-to-noise ratio (SNR) as faint vocalisations, often only partly visible on the spectrogram, or vocalisations that were clear and complete in the recording and spectrogram [following 32] (Suppl. Figs S1 and S2). Only vocalisations that were clear and complete were included in analysis. As a result, the included calls represented calls likely produced by the tagged whale or nearby individuals, while faint calls, likely to have been produced by more distant whales, were excluded from analysis. Calls, thought to function as communication signals^42^, were identified by horizontal bands in the spectrogram^43^ (Suppl. Fig. S1). Calls could contain a consistent click train component^42^ (Suppl. Fig. S1). Silence (no vocalisations, opposed to naturally occurring short pauses between vocalisations) was defined as an interval between consecutive vocalisations >24.5 s (see supplementary material for analysis; Suppl. Fig. S3). Call presence in the acoustic scene of the tagged whale was quantified as the proportion of time calls were recorded (no. seconds (s) of calling per minute).

### Visual data collection

Visual observations were conducted from the observation vessel at 6 m above sea level. Before attempts to tag started, the focal individual was selected from the whales present based upon easily identifiable features. The focal group was composed of all individuals in closer proximity to the focal individual and each other than to other individuals in the area^29^. Whenever the tagging vessel left the focal group without deploying a tag, or when a new individual became the focal (*i.e*. if the tagged whale was not the original focal individual), if possible, observations of the first focal whale were continued for 30 minutes to collect post-tagging data before starting a new follow.

We collected 6 group-level parameters to quantify the degree of aggregation, coordination and surface activity of the focal group: 1) group size, 2) number of individuals in the focal area (within 200 m of the focal whale), 3) distance to the nearest other group (categories: <50 m, 50–100 m, 100–200 m, 200–500 m, 500–1000 m, none in sight), 4) individual spacing (distance between individuals in the focal group; categories: very tight (<1 body lengths (BL)), tight (1–3 BL), loose (3–15 BL), very loose (>15 BL) and solitary (no other individual in focal area and/or distant from nearest neighbour), 5) surfacing synchrony (the proportion of individuals in the focal group surfacing during the surfacing period of the focal individual; categories: low (0–0.33), moderate (0.34–0.66) and high (0.67–1)) and 6) number of logging events (floating at the surface) in the focal group^29^.

Behavioural parameters were collected at 2-minute intervals when the focal whale was visible at the surface, or at first surfacing of the focal whale after a dive longer than 2 minutes. Group states were recorded by scanning the area around the focal individual or by traditional scan sampling, events were recorded using incident sampling^29^,^44^,^45^. Visual observation data were recorded using Logger 2000 software (IFAW).

### Analytical design

We investigated whether the behaviour of the focal group changed in response to the 6 experimental conditions using a Before-During-After analytical design. For the sonar exposure and killer whale playback phases, pre and post periods directly preceded and followed the exposure period (DUR). For the tagging phase, pre-tagging was defined as the 30-minute period prior to the start of tagging effort (launch of tagging vessel). The DUR period was defined as 15 minutes prior to tagging during tagging effort until 15 minutes after tag deployment, while the tag boat was still following the focal whale (Fig. 1). The moment of tag-on marks an exposure condition that is comparable between targeted whales (consistent, increasingly close approach followed by tag deployment), which is why this was used to anchor the DUR period. Tag deployment time was similar across whales (group behaviour: N = 4, median = 33 minutes, range: 25–35 minutes; vocal behaviour: N = 10, median = 35 minutes, range = 21–146 minutes). Post-tagging was defined as the first 30 minutes following the end of the tagging effort, after the tagging vessel left the whales and was more than 500 m away, or recovered. For the 2 records where no tag could be deployed, the DUR tagging period was started 20 minutes after the start of tagging effort, 15 minutes prior to the median tag deployment time. To enable quantification of and comparison to behavioural changes during baseline, each baseline phase was divided into simulated PRE, DUR and POST periods, starting from the start of the baseline phase (Fig. 1). PRE and POST periods had matching durations of approximately 30 minutes to their corresponding DUR period, or, for KW and noise control, 5–15 minute durations.

### Behavioural change score

First, for each PRE, DUR and POST period, we determined the maximum group size and number of individuals in the focal area, minimum distance category to other groups, average individual spacing and surfacing synchrony (see supplementary material for calculation), the number of loggings per individual per minute and the number of seconds per minute during which calls were recorded.

Second, behavioural changes between exposure periods within a session were quantified using two behavioural change scores 1) The difference between the DUR and the PRE period (PRE_DUR), representing the behavioural change occurring during exposure, compared to pre-exposure and 2) The difference between the POST and the PRE period (PRE_POST), indicating whether a behavioural change endured, or started, post-exposure (Fig. 1; see also^4^). We could not collect comparable vocalisation data during pre-tagging as no tag was deployed, so vocal parameters were excluded from change score analysis for the tagging phase.

In addition, we identified all DUR and POST periods during which the whales were silent (silent in 90–100% of experiment period) or highly vocal (vocal in 90–100% of experiment period). For tagging phases, the DUR period encompassed the 15 minutes following tag deployment (start recording) for this analysis.

### Statistical analysis of behavioural response

We compared behavioural changes during exposure to the three stimuli (tagging, sonar and KW) to changes during baseline behaviour and to each other. Changes during sonar and KW were also compared to changes during their respective controls (no-sonar and noise control). Differences in change scores from 16 focal groups were quantified using Generalised Estimating Equations (GEEs^46^;). Each group-level and vocalisation parameter was modelled as a Gaussian response variable against the factor covariates for *experiment condition* (tagging, sonar, KW, baseline, no-sonar control, noise control), *experiment period* (PRE_DUR, PRE_POST), *diving state* (predominantly shallow (S) or deep (D) diving in the PRE and DUR or POST periods: SS, SD, DD, DS) and the two-way interaction term *experiment condition:experiment period*. Diving state was included in the model to control for changes in group and vocal behaviour that may naturally occur between periods of deep and shallow diving^29^. To account for our repeated measures design, we specified a blocking unit (focal group ID) in the GEE, so that residuals were permitted to be correlated within, but assumed to be independent between blocking units. Models were run using the robust variance estimator^47^. Model selection was performed using a hypothesis-based backwards ANOVA (sequential Wald test). After running each GEE model and the ANOVA, the term with the largest p-value in the ANOVA model was removed, and the GEE model was rerun until all terms retained in the ANOVA were significant (p < 0.05). GEE models were run excluding outliers, defined as singular change scores of opposite sign and at least 5 times larger than the other change scores for the response variable. The GEE analyses were run using the package ‘geepack’^48^ in R, version 2.14.1^49^.

We expected to find a significant effect of *experiment condition* on the response variable if changes in behaviour during tagging, sonar or KW were different from baseline or the no-sonar and noise controls. If *experiment condition* was retained in the model, we determined the significance of differences between pairs of conditions (e.g. sonar vs. baseline) using the p-values for differences between *experiment condition* factor levels generated by the final GEE model. We expected a significant effect of *experimental period* on the response variable if groups changed their behaviour during exposure and returned to pre-exposure behaviour after the end of exposure, or if they started to change behaviour during post-exposure.

### Ethics statement

All research activities were carried out under permits issued by the Norwegian Animal Research Authority (permit no. S-2007/61201), in compliance with ethical and local use of animals in experimentation. The research protocol was approved by the University of St Andrews Animal Welfare and Ethics Committee and Woods Hole Oceanographic Institution’s Animal Care and Use Committee.

## Additional Information

**How to cite this article**: Visser, F. *et al*. Disturbance-specific social responses in long-finned pilot whales, *Globicephala melas*. *Sci. Rep*. **6**, 28641; doi: 10.1038/srep28641 (2016).

## Supplementary Material

Supplementary Information

## Figures and Tables

**Table 1:** Summary of experiments with long-finned pilot whales.

				No. records per Experiment condition (PRE_DUR; PRE_POST)					
TAG ID	Date	Ind. class	Data type	TAG	BASE	SONAR	no- sonar ctrl	KW	noise ctrl
gm08_150c	29-05-2008	MC	V	0;0^*^	0;0	2;2			
gm08_154d	02-06-2008	MC	V	0;0^*^	1;0	2;2			
gm08_156a	04-06-2008	MC	V	0;0^*^					
gm08_159a	07-06-2009	L	V	0;0^*^	1;0	2;2	1;1	2;2	
gm09_137a	17-05-2009	MC	GV	1;10;0^*^	4;44;4				
gm09_138a	18-05-2009	M	GV	1;10;0^*^	1;11;1	3;33;3	1;11;1		
gm09_156b	05-06-2009	L	GV	0;00;0^*^	3;23;2	3;33;3	1;11;1	2;1	
no tag (a)	05-06-2009	M	G	1;0					
LpW_10pm1N	24-05-2010	L	G	0;0	1;0			2;2	2;2
gm10_143a	23-05-2010	L	GV	0;00;0^*^	5;55;5				
gm10_152b	01-06-2010	M	GV	0;00;0^*^	1;01;0				
gm10_157a	06-06-2010	L	G	1;1					
gm10_157b	06-06-2010	MC	GV	0;0^*^	5;57;6				2;12;1
gm10_158a	07-06-2010	M	V	0;0^*^					
gm10_158d	07-06-2010	M	GV	1;10;0^*^	1;11;1			2;22;2	2;02;2
no tag (b)	07-06-2010	M	G	1;1					
TOTAL			G (86)V (88)	6;50;0	21;1824;19	6;612;12	2;23;3	6;54;4	6;34;3

TAG ID = identification number of tagged whale for each experiment. No tag: no individual tagged. Individual class: L = large adult, M = medium-sized adult, C = individual consistently associated with calf. Data type: G = group-level behaviour, V = vocal behaviour. TAG = tagging, BASE = baseline, KW = killer whale sounds playback. Ctrl = control (Fig. 2). 0: PRE, DUR and/or POST period not (fully) recorded (Fig. 1). Empty cell: condition not present. No sonar experiments were conducted in 2010. ^*^Tagging phase vocal data included in analysis of silent/fully vocal periods; N = 12 DUR and 12 POST periods.

## References

[b1] Caro T. Anti-predator defences in birds and mammals. 592 pp (University of Cambridge Press), (2005).

[b2] Lima S. L. & Dill L. M. Behavioural decisions made under the risk of predation: a review and prospectus. Can. J. Zool. 68, 619–640 (1990).

[b3] Hemmi J. M. & Pfeil A. A multi-stage anti-predator response increases information on predation risk. J. Exp. Biol. 213, 1484–1489 (2010). 20400633 10.1242/jeb.039925

[b4] Curé . Predator sound playbacks reveal strong avoidance responses in a fight strategist baleen whale. Mar. Ecol. Prog. Ser. 526, 267–282 (2015).

[b5] Weston M. A., McLeod E. M., Blumstein D. T. & Guay P. J. A review of flight-initiation distances and their application to managing disturbance to Australian birds. Emu 112, 269–286 (2012).

[b6] Deecke V. B., Slater P. J. B. & Ford J. K. B. Selective habituation shapes acoustic predator recognition in harbour seals. Nature 420, 171–173 (2002). 12432391 10.1038/nature01030

[b7] McComb K., Shannon G., Sayialel K. N. & Moss C. Elephants can determine ethnicity, gender, and age from acoustic cues in human voices. PNAS doi: 10.1073/pnas.1321543111 (2014). PMC3986134 24616492

[b8] Elgar M. A. Predator vigilance and group size in mammals and birds: a critical review of the empirical evidence. Biol. Rev. 64, 13–33 (1989). 2655726 10.1111/j.1469-185x.1989.tb00636.x

[b9] Roth E. D. & Johnson J. A. Size-based variation in anti-predator behaviour within a snake (*Agkistrodon piscivorus*) population. Behav. Ecol. 15, 365–370 (2004).

[b10] Frid A. & Dill L. Human-caused disturbance stimuli as a form of predation risk. Conserv. Ecol. 6, 11 (2002).

[b11] Blumstein D. T. Developing an evolutionary ecology of fear: how life history and natural history traits affect disturbance tolerance in birds. Anim. Behav. 71, 389–399 (2006).

[b12] Marshall H. H., Carter A. J., Rowcliffe J. M. & Cowlishaw G. Linking social foraging behaviour with individual time budgets and emergent group-level phenomena. Anim. Behav. 84, 1295–1305 (2012).

[b13] Manser M. B. The acoustic structure of suricates’ alarm calls varies with predator type and the level of response urgency. Proc. R. Soc. Lond. B. 268, 2315–2324 (2001). 10.1098/rspb.2001.1773 PMC1088882 11703871

[b14] Hill R. A. & Dunbar R. I. M. An evaluation of the roles of predation rate and predation risk as selective pressures on primate grouping behaviour. Behaviour 135, 411–430 (1998).

[b15] Richardson W. J., Greene C. R., Malme C. I. & Thomson D. H. Marine mammals and noise 558 pp (Academic Press, 1995).

[b16] Tyack P. L. Implications for marine mammals of large-scale changes in the marine acoustic environment. J. Mamm. 89, 549–558 (2008).

[b17] Bejder L. . Decline in relative abundance of bottlenose dolphins exposed to long-term disturbance. Conserv. Biol. 20, 791–1798 (2006). 10.1111/j.1523-1739.2006.00540.x 17181814

[b18] Pirotta E. . Vessel noise affects beaked whale behavior: results of a dedicated acoustic response study. PLoS One 7, e42535 (2012). 22880022 10.1371/journal.pone.0042535 PMC3411812

[b19] New L. F., Moretti D. J., Hooker S. K., Costa D. P. & Simmons S. E. Using energetic models to investigate the survival and reproduction of beaked whales (family *Ziphiidae*). PloS One 8, e68725 (2013). 23874737 10.1371/journal.pone.0068725 PMC3714291

[b20] Miller P. J. O. . Dose-response relationships for the onset of avoidance of sonar by free-ranging killer whales. J. Acoust. Soc. Amer. 135, 975–993 (2014). 25234905 10.1121/1.4861346

[b21] Williams R. & Ashe E. Killer whale evasive tactics vary with boat number. J. Zool. 272, 390–397 (2007).

[b22] Miller P. J. O. . The severity of behavioral changes observed during experimental exposures of killer (*Orcinus orca*), long-finned pilot (*Globicephala melas*), and sperm (*Physeter macrocephalus*) whales to naval sonar. Aquat. Mamm. 38, 362–401 (2012).

[b23] Harris C. M. . Dose response severity functions for acoustic disturbance in cetaceans using recurrent event survival analysis. Ecosphere 6, 1–14 (2016).

[b24] DeRuiter S. L. . First direct measurements of behavioural responses by Cuvier’s beaked whales to mid-frequency active sonar. Biol. Lett. 9, 20130223 (2013). 23825085 10.1098/rsbl.2013.0223 PMC3730631

[b25] Antunes R. . High response thresholds for avoidance of sonar by free-ranging long-finned pilot whales *Globicephala melas*. Mar Pollut. Bull. 32, 323–346 (2014). 10.1016/j.marpolbul.2014.03.056 24820645

[b26] Amos B. Social structure of pilot whales revealed by analytical DNA profiling. Science 260, 670–672 (1993). 8480176 10.1126/science.8480176

[b27] Curé C. . Pilot whales attracted to killer whale sounds: acoustically-mediated interspecific interactions in cetaceans. PLoS One 7, e52201 (2012). 23300613 10.1371/journal.pone.0052201 PMC3530591

[b28] DeStephanis R. . Mobbing-like behavior by pilot whales towards killer whales: a response to resource competition or perceived predation risk? Acta Ecolog. doi: 10.1007/s10211-014-0189-1 (2014).

[b29] Visser F. . The social context of individual foraging behaviour in long finned pilot whales. Behaviour 151, 1453–1477 (2014).

[b30] Taruski A. G. The whistle repertiore of the North Atlantic pilot whale (Globicephala melaena) and its relationship to behavior and environment. in Behavior of marine mammals, Vol 3. (eds Winn H. E. & Olla B. L.) 345–368 (Plenum Press, 1979).

[b31] Rendell L. E. & Gordon J. C. D. Vocal response of long-finned pilot whales (*Globicephala melas*) to military sonar in the Ligurian Sea. Mar. Mammal Sci. 15, 198–204 (1999).

[b32] Alves A. C., Antunes R. N., Bird A., Tyack P. L. & Miller P. J. O. Vocal matching of naval sonar signals by long-finned pilot whales *Globicephala melas*. Mar. Mammal Sci. doi: 101111/mms12099 (2014).

[b33] Miller P. J. O. . First indications that northern bottlenose whales are sensitive to behavioural disturbance from anthropogenic noise. Roy. Soc. Open Sci. 2, 140484 (2015). 26543576 10.1098/rsos.140484 PMC4632540

[b34] Sivle L. D. . Severity of expert-identified behavioural responses of humpback whale, minke whale and Northern bottlenose whale to naval sonar. Aquat. Mamm. doi: 10.1578/AM.41.4.2015.xxx (2015).

[b35] Sivle L. D. . Changes in dive behavior during sonar exposure in killer whales, pilot whales and sperm whales. Frontiers Aquat. Physiol. doi: 10.3389/fphys.2012.00400 (2012). PMC3468818 23087648

[b36] Wensveen P. J. . How effectively do synchronised avoidance and other response strategies of pilot whales reduce sounds exposure from naval sonar? Mar. Environ. Res. 106, 86–81 (2015). 10.1016/j.marenvres.2015.02.005 25795075

[b37] Jensen F. H., Perez J. M., Johnson M., Soto N. A. & Madsen P. T. Calling under pressure: short-finned pilot whales make social calls during deep foraging dives. Proc. R. Soc. Lond. B. 278, 3017–3025 (2011). 10.1098/rspb.2010.2604 PMC3158928 21345867

[b38] Weihs D. & Webb P. W. Optimal avoidance and evasion tactics in predator-prey interactions. J. Theor. Biol. 106, 189–206 (1984).

[b39] Miller P. J. O. . The 3S experiments: studying the behavioural effects of naval sonar on killer whales (*Orcinus orca*), sperm whales (*Physeter macrocepha*lus), and long-finned pilot whales (*Globicephala melas*) in Norwegian waters. Scottish Oceans Inst. Tech. Rept. SOI-2011-001 (2011).

[b40] Johnson M. P. & Tyack P. L. A digital acoustic recording tag for measuring the response of wild marine mammals to sound. IEEE J. Ocean. Engin. 28, 3–12 (2003).

[b41] Aoki K., Sakai M., Miller P. J. O., Visser F. & Sato K. Body contact and synchronous diving in long-finned pilot whales. Behav. Proc. 99, 12–20 (2013). 10.1016/j.beproc.2013.06.002 23769937

[b42] Sayigh L., Quick N., Hastie G. & Tyack P. Repeated call types in short-finned pilot whales, *Globicephala macrorhynchus*. Mar. Mamm. Sci. 29, 312–324 (2012).

[b43] Watkins W. A. The harmonic interval: fact or artifact in spectral analysis of pulse trains. In Marine Bioacoustics, Vol. 2. (ed. Tavolga W. N.) 15–43 (Pergamon Press, 1976).

[b44] Altmann J. Observational study of behavior: sampling methods. Behaviour 49, 227–267 (1974). 4597405 10.1163/156853974x00534

[b45] Mann J. Behavioral sampling methods for cetaceans: a review and critique. Mar. Mammal Sci. 15, 102–122 (1999).

[b46] Hardin J. & Hilbe J. In Generalized Estimating Equations (Chapman and Hall/CRC Press), (2003).

[b47] Zorn C. Comparing GEE and robust standard errors for conditionally dependent data. Polit. Res. Quart. 59, 329–341 (2006).

[b48] Højsgaard S., Halekoh U. & Yan J. The R Package geepack for Generalized Estimating Equations. J. Stat. Software 15, 1–11 (2006).

[b49] R Development Core Team. R: *A language and environment for statistical computing*. R Foundation for Statistical Computing, Vienna, Austria. ISBN 3-900051-07-0, http://www.R-project.org (2011).

