# Supplementary material for: Disturbance-specific social responses in long-finned pilot whales, Globicephala melas
Source: Sci Rep. 2016 Jun 29;6:28641. doi: 10.1038/srep28641 (PMC4926103; doi:10.1038/srep28641)
Supplement: Supplementary Information [file srep28641-s1.pdf]

Electronic Supplementary material

Electronic supplementary material for the manuscript: *'Disturbance-specific social responses in long-finned pilot whales, Globicephala melas'* by Fleur Visser, Charlotte Curé, Petter Kvadsheim, Frans-Peter Lam, Peter Tyack and Patrick Miller.

Methods

Sound stimuli

Naval sonar signals were transmitted as 1 s signals at 20 s intervals. The signals were 1-2 or 6-7 kHz, respectively transmitted from 158 /152 dB re 1µPa m (rms) to full power at 214 /199 dB re 1µPa m during the 10 minutes of ramp-up. The combination of ramp-up of source level and source vessel approach resulted in an escalation of the sound pressure level (SPL) received by the whales [39]. Maximum received SPL levels per session ranged from 150-180 dB re 1µPa, within a range of 68-180 dB re 1µPa for all received signals [25].

Killer whale playback transmissions consisted of DTAG recordings of sounds (calls, clicks and tailslaps) of killer whales (*Orcinus orca*) feeding on herring in northern Norway. The noise control stimuli consisted of sequences of background noise in the killer whale sound recordings (0.5-10 kHz), amplified to the same average source level as the killer whale sounds (140-155 dB re 1µPa m). One of three different versions of each stimulus type was randomly selected for each session. The killer whale playback protocol is further detailed in Curé et al. [27].

Defining silence

We used a log-frequency analysis of time intervals between all consecutive calls and click series to determine the duration threshold between silent periods and periods of vocalisations with naturally occurring pauses (baseline phase data only; [50,51]). We fitted a one-process model (no distinction between pauses and silent periods), a two-process model (pauses + silent periods) and a three-process model (pauses + 2 categories of silent periods) to the log-frequency distribution. The best of these three models was selected using the Akaike Information Criterion (AIC). Log-frequency analysis of the intervals between vocalisations indicated a two-order process, with a threshold duration of 24.5 s of continuous silence distinguishing between vocal periods with naturally occurring pauses and silent periods (Fig. S3).

50. Sibly, R.M., Nott, H.M.R. & Fletcher, D.J. Splitting behaviour into bouts. *Anim. Behav.* **39,** 63-69 (1990).

51. Miller, P.J.O., Shapiro, A.D., Tyack, P.L & Solow, R. Call-type matching in vocal exchanges of free-ranging resident killer whales, *Orcinus orca*. *Anim. Behav.* **67**, 1099-1107 (2004).

Behavioural change score

Calculation average individual spacing and synchrony

To calculate average individual spacing and surfacing synchrony, we determined which parameter category was predominantly recorded during baseline. The remaining categories were then quantified as +1, or -1 depending on whether they represented an increase or a decrease in spacing or synchrony with respect

to the predominant category, quantified as 0 (neutral). Tight individual spacing and moderate synchrony were most commonly observed, representing 56% and 61% of records. Thus, e.g. synchrony records were quantified as low = -1, moderate = 0 and high = 1.

Supplementary Figure S1.

Spectrogram examples of clear and complete pilot whale vocalisations included in analysis. (A) Call with multiple harmonics (example 1). (B) Call with multiple harmonics (example 2). (C) Slow echolocation click series followed by a short pause and a fast click series. Slow and fast click series made in foraging context as part of the prey search (slow) and capture attempt (fast) phases of bio-sonar based foraging. (D) Call with a fast click series component. These were defined as calls with clicks series that showed a consistent duration, shape and timing with respect to the start of the same frequency modulated call (if repeated), or when transition between frequency modulated and fast click component within one and the same call was apparent [42]. Spectrogram settings: Blackman-Harris window, FFT length: 4096, 75% overlap, 132 dB dynamic range. SNR (dB) A) 30.5, B) 37.0, C) click: 7.9, D) 16.5 (2nd call).

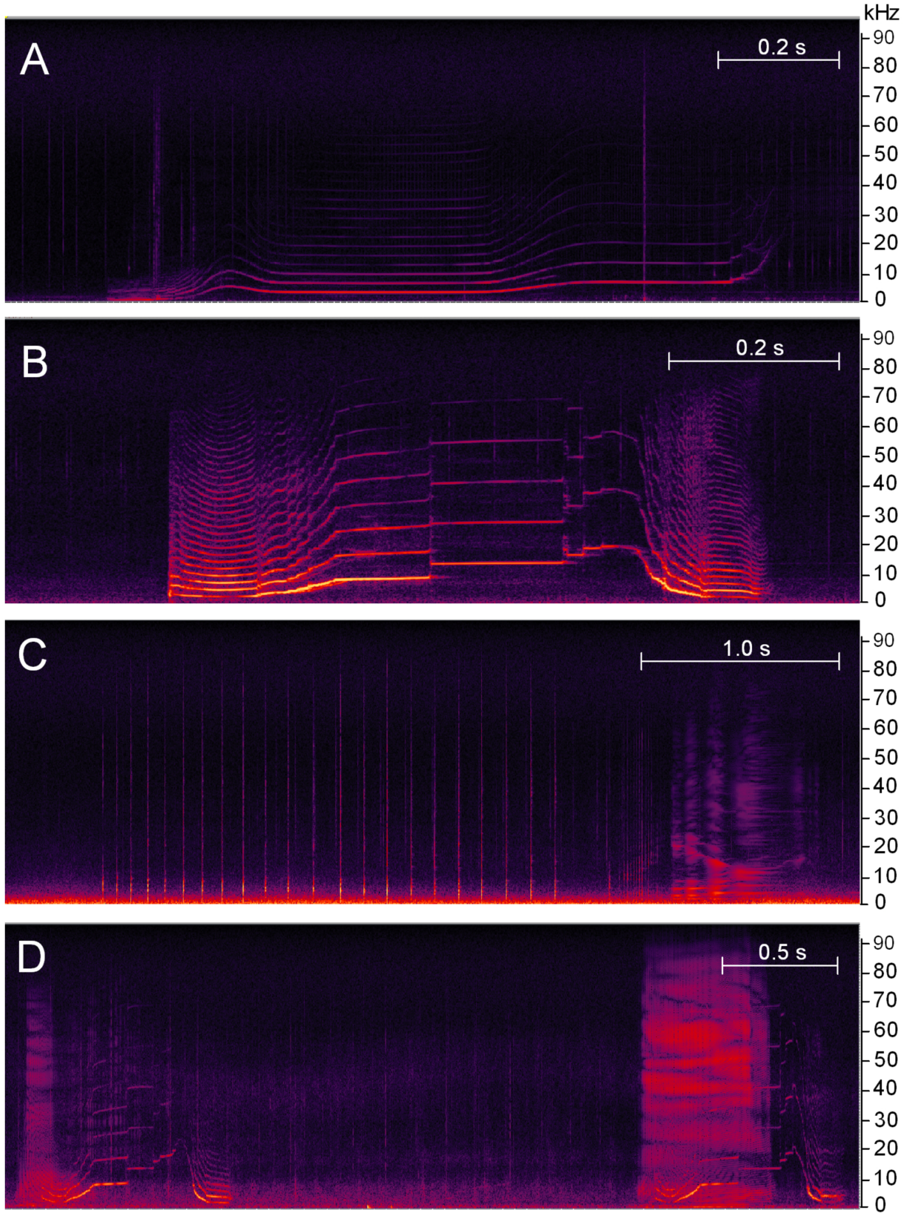

Supplementary Figure S2.

Spectrogram examples of faint pilot whale vocalisations, excluded from analysis. (A) Call with multiple harmonics (example 1). (B) Call with multiple harmonics (example 2). (C) Slow echolocation click series. Spectrogram settings as in supplementary Fig. S1. SNR (dB): A) -5.7, B) 0.1, C) click: 2.5.

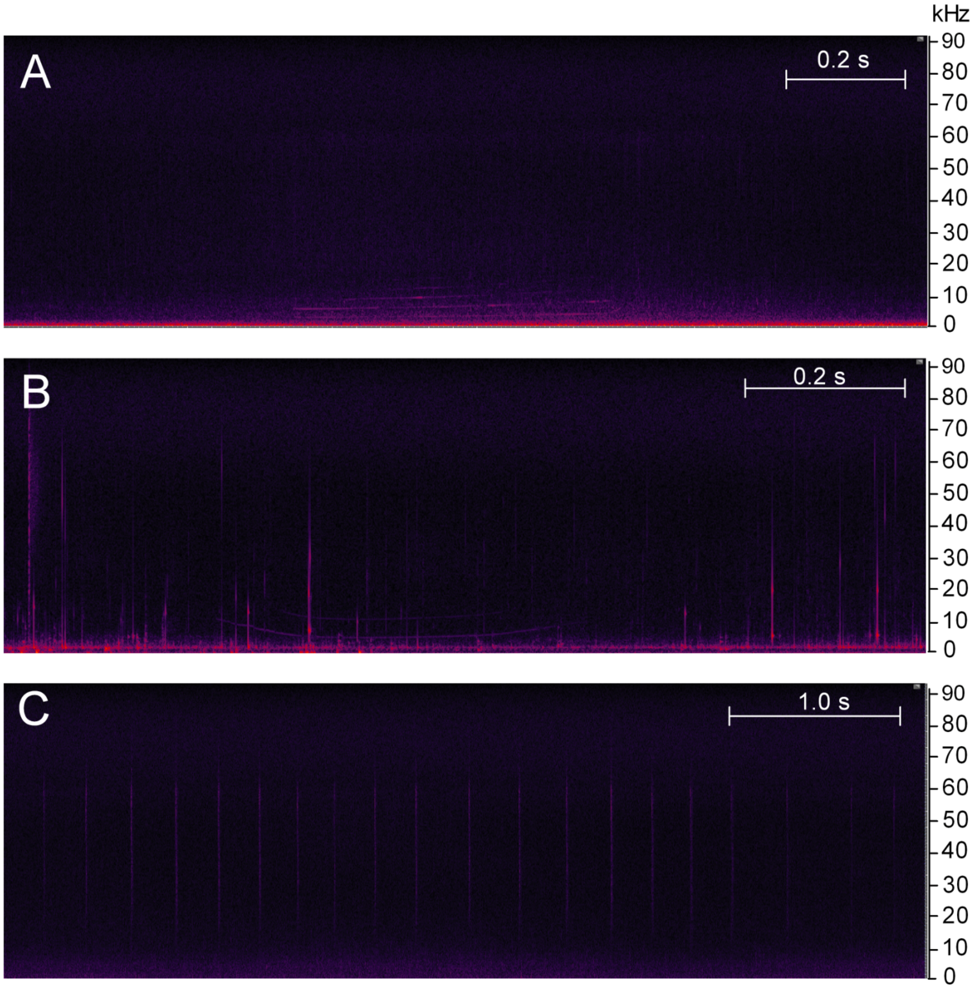

Supplementary Figure S3. *Log-frequency distribution of intervals between vocalisations during baseline. The vertical dashed line indicates the interval length that was used to distinguish between vocal periods with naturally occurring pauses (intervals < 24.5 s) and silent periods (intervals > 24.5 seconds). AIC two-process model: 32597; three-process model: 32679; one-process model: 37903.*

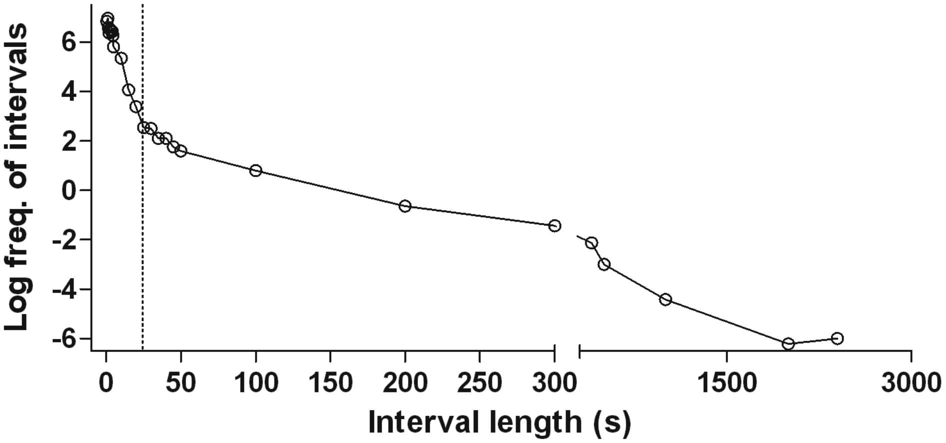

Supplementary Table S1*. Wald statistic p-values for all covariates in A) the 6 GEE models for group-level behaviour and B) the GEE model for calling behaviour. Bold text: significant at p<0.05. Parameter estimates and standard errors per factor level for retained covariates are given in Table S2.*

	Condition	*Diving state*	*Period*	*Condition:Period*
A. Group behaviour				
Group size	***<0.0001***	***0.002***	*0.4**	***<0.0001***
No. in focal area	***<0.0001***	***0.04***	*0.22*	*0.28*
Dist. to nearest other group	***<0.0001***	*0.55*	*0.13*	*0.50*
Individual spacing	***<0.0001***	***0.03***	*0.6**	***<0.0001***
Surfacing synchrony	***<0.0001***	*0.32*	*0.14*	*0.46*
No. loggings/ind./minute	***0.002***	*0.12*	*0.62**	***<0.0001***
B. Calling behaviour				
Call rate (no. s/minute)	*0.18**	*0.12*	*0.52**	***0.0002***

**Retained in best model because included in significant two-way interaction term.*

Supplementary Table S2. *Coefficients ± standard error and p-values for retained covariates in the best-fit Generalised Estimating Equation model (Table S1) investigating differences in group and calling behaviour between experiment conditions, experiment periods, and diving states. Coefficients represent the difference between the factor level and the reference factor level. Conditions: tagging (TAG), baseline (BASE), sonar, no-sonar control, killer whale playback (KW) and noise control. D = deep diving, S = shallow diving in PRE and DUR or POST of the experiment period (e.g. DS = deep diving in PRE, shallow diving in DUR for PRE_DUR experiment period). Bold text: significant difference between factor level and reference factor level at p<0.05.*

Re-sponse	Sign. effect	Factor level	Reference factor level<br>BASE	TAG	SONAR	No-sonar control	Noise control
Group size	Condition	TAG	**7.6±2.1 p=0.0003**				
		SONAR	**13.4±1.3 p=<0.0001**	**5.8±2.4 p=0.01**		11.6±7.8 p=0.14	
		KW	**4.5±1.7 p=0.007**	-3.1±2.9 p=0.28	**-8.9±1.9 p=<0.0001**		2.0±1.5 p=0.18
			PRE_DUR				
	Period	PRE_ POST	0.9±0.9 p=0.3				
			DD	SS	SD		
	Diving state	DS	2.8±3.1 p=0.4	-1.8±2.4 p=0.45	0.8±2.0 p=0.7		
		SD	2.0±2.9 p=0.5	**-2.6±0.8 p=0.002**			
		SS	4.6±2.9 p=0.1				
			BASE: PERIOD	TAG: PERIOD	SONAR: PERIOD	SONAR CTRL: PERIOD	NOISE CTRL: PERIOD
	Condition :Period	TAG: PERIOD	-2.0±2.2 p=0.36				
		SONAR: PERIOD	-2.7±2.2 p=0.23	-0.63±3.1 p=0.84		**-9.4±4.8 p=0.046**	
		KW: PERIOD	4.1±4.3 p=0.34	6.1±4.6 p=0.19	6.7±4.4 p=0.13		6.5±4.4 p=0.14

			BASE	TAG	SONAR	NO-SONAR CTRL	NOISE CTRL
No. in focal area	Condition	TAG	2.6±3.3 p=0.4				
		SONAR	6.2±3.8 p=0.1	3.6±4.6 p=0.4		4.5±3.4 p=0.18	
		KW	**17.8±5.1 p=0.0005**	**15.3±7.3 p=0.036**	11.7±8.1 p=0.2		**19.3±7.5 p=0.0097**
			DD	SS	SD		
	Diving state	DS	-2.8±3.3 p=0.4	**-5.0±1.9 p=0.01**	1.64±3.6 p=0.65		
		SD	-4.4±6.1 p=0.5	-6.6±3.9 p=0.09			
		SS	2.2±4.1 p=0.6				
			BASE	TAG	SONAR	NO-SONAR CTRL	NOISE CTRL
Dist. to other gr.	Condition	TAG	**1.7±0.25 p=<0.0001**				
		SONAR	0.17±0.12 p=0.16	**-1.5±0.324 p=<0.0001**		-0.8±1.38 p=0.6	
		KW	**-1.01±0.20 p=<0.0001**	**-2.7±0.29 p=<0.0001**	**-1.2±0.15 p=<0.0001**		**-1.9±0.5 p=0.0003**
			BASE	TAG	SONAR	NO-SONAR CTRL	NOISE CTRL
Ind. spacing	Condition	TAG	-0.22±0.14 p=0.12				
		SONAR	-0.31±0.39 p=0.43	0.09±0.39 p=0.8		- 0.36±0.3	
		KW	0.20±0.12 p=0.08	**0.42±0.20 p=0.03**	0.51±0.41 p=0.21		-0.02±0.12 p=0.9
			PRE_DUR				
	Period	PRE_ POST	-0.2±0.23 p=0.40				
			DD	SS	SD		
	Diving state	DS	0.03±0.17 p=0.88	**-0.38±0.17 p=0.03**	0.06±0.22 p=0.8		
		SD	0.08±0.22 p=0.71	**-0.32±0.16 p=0.045**			
		SS	**0.41±0.17 p=0.02**				

			BASE: PERIOD	TAG: PERIOD	SONAR: PERIOD	NO-SONAR CTRL: PERIOD	NOISE CTRL: PERIOD
	Condition :Period	TAG: PERIOD	-0.28±0.22 p=0.2				
		SONAR: PERIOD	0.06±0.18 p=0.8	0.34±0.33 p=0.30		0.29±0.5 1 p=0.57	
		KW: PERIOD	**-0.17±0.08 p=0.03**	0.11±0.23 p=0.6	-0.23±0.24 p=0.34		**0.24±0.09 p=0.01**
			BASE	TAG	SONAR	NO-SONAR CTRL	NOISE CTRL
Surf. synch	Condition	TAG	**0.35±0.14 p=0.01**				
		SONAR	0.02±0.09 p=0.9	**-0.33±0.12 p=0.007**		0.38±0.2 7p=0.1	
		KW	-0.02±0.06 p=0.8	**-0.37±0.14 p=0.007**	-0.04±0.11 p=0.74		-0.24±0.16 p=0.12
			BASE	TAG	SONAR	NO-SONAR CTRL	NOISE CTRL
No. log/ ind./min	Condition	TAG	-0.03±0.02 p=0.2				
		SONAR	0.015±0.01 p=0.3	0.04±0.02 p=0.053		0.03±0.0 1	
		KW	-0.01±0.009 p=0.1	0.014±0.017 p=0.4	-0.03±0.02 p=0.1		-0.01±0.01 p=0.23
			PRE_DUR				
	Period	PRE_ POST	-0.003±0.006 p=0.6				
			BASE: PERIOD	TAG: PERIOD	SONAR: PERIOD	SONAR CTRL: PERIOD	KW CTRL: PERIOD
	Condition :Period	TAG: PERIOD	-0.008±0.02 p=0.6				
		SONAR: PERIOD	0.05±0.04 p=0.13	**0.06±0.03 p=0.03**		0.05±0.0 3 p=0.12	
		KW: PERIOD	0.001±0.03 p=0.98	0.009±0.03 p=0.8	-0.05±0.03 p=0.07		- 0.003±0.0 3 p=0.9
Calling (no. s/min)	Condition		BASE	SONAR	SONAR CTRL	KW CTRL	
		SONAR	1.2±1.05 p=0.3		-0.07±1.8 p=0.97		
		KW	11.6±5.3 p=0.03*	10.5±5.0 p=0.035*		11.9±5.2 p=0.02*	

Period		PRE_POST			
	PRE_ DUR	-0.07±0.9 p=0.9			
		BASE: PERIOD	SONAR: PERIOD	SONAR CTRL: PERIOD	KW CTRL: PERIOD
Condition :Period	SONAR: PERIOD	1.2±1.1 p=0.28		0.17±1.5 p=0.9	
	KW: PERIOD	**-7.7±3.9 p=0.047**	**-8.9±3.7 p=0.016**		**-8.7±4.3 p=0.04**

*Significant relation as part of two-way interaction term (single-term significance not interpreted).
